# Pattern of sexually transmitted infections and performance of syndromic management against etiological diagnosis in patients attending the sexually transmitted infection clinic of a tertiary care hospital

**DOI:** 10.4103/2589-0557.74998

**Published:** 2010

**Authors:** Shilpee Choudhry, V. G. Ramachandran, Shukla Das, S. N. Bhattacharya, Narendra Singh Mogha

**Affiliations:** Department of Microbiology, UCMS & GTB Hospital, Dilshad Garden, Delhi, India; 1 Department of Dermatology and STD, UCMS & GTB Hospital, Dilshad Garden, Delhi, India

**Keywords:** Genital discharge syndrome, genital ulcer syndrome, genital wart, HIV, sexually transmitted infection, syndromic approach

## Abstract

**Background and Objectives:**

The availability of baseline information on the epidemiology of sexually transmitted infections (STIs) and other associated risk behaviors is essential for designing, implementing, and monitoring successful targeted interventions. Also, continuous analysis of risk assessment and prevalence-based screening studies are necessary to evaluate and monitor the performance of syndromic management. The aim of the present study was to document the pattern of common STIs and to evaluate the performance of syndromic case management against their laboratory diagnoses.

**Materials and Methods:**

Three hundred consecutive patients who attended the STI clinic of a tertiary care hospital at Delhi, with one or more of the complaints as enunciated by WHO in its syndromic approach for the diagnosis of STIs, were included as subjects. Detailed history, demographical data, and clinical features were recorded and screened for common STIs by standard microbiological methods.

**Results:**

The mean age was 24 years and most of the male patients were promiscuous and had contact with commercial sex workers (CSWs 63.9%). Majority came with the complaint of genital discharge (63 males; 54 females) followed by genital ulcer (61 males; 30 females). Genital herpes accounted for the maximum number of STI (86/300) followed by syphilis (71/300). The sensitivity of genital discharge syndrome (GDS) was high for Neisseria gonorrhoeae and Chlamydia trachomatis (96% and 91%, respectively) while specificity was low (76% and 72%, respectively). The sensitivity of genital ulcer syndrome for herpes simplex virus-2 (HSV-2) and Treponema pallidum was 82.65% and 81.2%, respectively, while specificity reached 99% approximately.

**Conclusions:**

Viral STIs constitute the major burden of the STI clinic and enhance the susceptibility of an individual to acquire or transmit HIV through sexual contact. Syndromic algorithms have some shortcomings, and they need to be periodically reviewed and adapted to the epidemiological patterns of STI in a given setting.

## INTRODUCTION

Sexually transmitted infections (STIs), including human immunodeficiency virus (HIV), continue to present major health, social, and economic problems in the developing world, leading to considerable morbidity, mortality, and stigma. The prevalence rates apparently are far higher in developing countries where STI treatment is less accessible.[1]

Most of the STIs, both ulcerative and nonulcerative, are prevalent in India and constitute one of the major public health problems. Their profile varies with changes in socioeconomic, cultural, geographic, and environmental factors prevalent in different parts of the country.[2–6] However, due to lack of adequate laboratory infrastructure in the country, information regarding the profile of STIs relies essentially on syndromic diagnosis. Hence there is very limited data of laboratory-proven STIs.[7,8] However, the availability of baseline information on the epidemiology of STIs and other associated risk behaviors remains essential for the designing, implementing, and monitoring successful targeted interventions.[9,10]

The World Health Organization (WHO) has placed emphasis on syndromic approach for case measurement and management, particularly in high-prevalence areas having inadequate laboratory facilities, trained staff, and transport facilities.[11] Though the syndromically diagnosed STI has many limitations, continuous analysis of risk assessment and prevalence-based screening studies are necessary to evaluate and monitor the performance of syndromic management.[12]

The aim of the present study was to document the pattern of common STIs in patients attending the STI clinic of a tertiary care hospital, and to evaluate the performance of syndromic case management against their laboratory diagnoses.

## MATERIALS AND METHODS

Three hundred consecutive patients from April 2007 to December 2008, who attended the STI clinic of a tertiary care hospital in Delhi, with one or more of the complaints as enunciated by WHO in its syndromic approach for the diagnosis of STI[13] were included as subjects. Followed up patients and asymptomatic patients were excluded from the study. Detailed history, demographical data, and clinical features were recorded from all the patients. All patients were managed on the basis of algorithms of the syndromic approach at the peripheral health center (PHC) level recommended by national AIDS control organization (NACO), India, after carrying out risk assessment.[14] All were screened for common STIs by standard microbiological methods.[15]

Urethral and endocervical swabs were collected from males and females, respectively, and subjected to direct examination by Gram staining and culture plate inoculation at the site of sample collection. A presumptive diagnosis of gonococcal infection was made on observing polymorphonuclear leucocytes (PMNLs) with Gram-negative intracellular diplococci (ICDC). If the smear showed five or more PMNLs in the absence of Gram-negative ICDC, a presumptive diagnosis of nongonococcal urethritis (NGU) was made in men.[16] For the isolation of *Neisseria gonorrhoeae*, swabs were directly inoculated on the chocolate agar plate containing vancomycin, colistin, and amphotericin-B and incubated in 5–10% carbon dioxide for 24–48 h. Isolates were identified as *N. gonorrhoeae* on the basis of colony morphology, Gram staining, oxidase test, and rapid carbohydrate utilization test (RCUT).[17]

Normal saline wet mount examinations were done to detect motile trophozoites of *Trichomonas vaginalis* and yeast cells for *Candida* infection. For the isolation of *Candida*, urethral/cervical discharge was inoculated on Sabouraud dextrose agar and identification was done by standard mycological techniques.[15]

A direct smear was made from the ulcer, if any, and subjected to direct examination by Gram staining and Leishman staining for the presence of multinucleated giant cells, shoals of fish bacilli, or safety pin-appearing bacilli to detect herpes simplex virus (HSV), *Hemophilus ducreyi*, and *Calymmatobacterium granulomatis*, respectively.[15]

Ten milliliters venous blood (without anticoagulant) was collected aseptically from all patients. Sera were separated and stored at −20°C in screw-capped glass tubes. HSV-2 IgM antibody in patients’ sera was detected by the Ridascreen HSV-2 IgM (K5231, Germany) kit according to the manufacturer’s instructions. It is an indirect enzyme immunoassay for the semiquantitative estimation of IgM antibodies against the HSV type-2 in human serum. HSV-1 IgM antibody was also detected by Meddens Diagnostics herpes simplex virus IgM *µ*; capture EIA (REF 4051, The Netherlands). It is an antibody class capture immunosorbant assay for the detection of HSV IgM in human serum. Sera were also tested for antibodies of other STIs namely hepatitis B virus (HBV; 0003463 Hepalisa kit) and hepatitis C virus (HCV; third-generation HCV Microlisa kit, India) by ELISA and *Treponema pallidum* by Venereal Disease Research Laboratory (VDRL) test (an antigen from the serologist of Kolkata, Government of India) followed by the *T. pallidum* hemagglutination test (TPHA; Plasmatec TPHA test kit, Hansard Diagnostic, UK). Antigen detection for *Chlamydia trachomatis* in the genital swab of all the patients was performed by the Bio-Rad Chlamydia Microplate EIA (31189 United States) kit. All patients were tested for HIV by ELISA/rapid tests, using WHO-approved kits, following NACO guidelines, after pretest counseling and written informed consent, followed by post-test counseling.[18] Genital wart and molluscum contagiosum was detected clinically.

The proportions were calculated for various syndromes and disease prevalence. Sensitivity, specificity, negative predictive value (NPV), and positive predictive value (PPV) of various syndromes were calculated. Confidence intervals for STI prevalence were calculated for future monitoring.

## RESULTS

### Sociodemographic profile

The age of patients ranged from 15 to 53 years, the mean age being 24 years, and 62% of them were in the age group of 20–30 years. Sixty-four percent of the patients were male, and the male-to-female ratio was 2:1. Majority of the male patients (53.12%) were educated to the level of middle school while 50% of the females were illiterate. Sixty percent of the patients were married at the time of presentation and all but six of them were cohabiting with their spouse. Only 19% of them reported regular use of condom. Most of the patients (67%) had first sexual exposure between the ages of 19–25 years while 31% of the males and 22.22% of the females had first sexual contact at or before 18 years of age. Sixty-five percent of males had multiple sexual partners in the past 6 months and 63.9% had contact with commercial sex workers (CSWs). In contrast, most of the female patients (83.3%) had one sexual partner (husband in 80% of them).

### Prevalence of sexually transmitted infection syndromes

Majority of the patients came with the complaints of discharge (63 males; 54 females) followed by genital ulcer (61 males; 30 females) as shown in Table 1. Twelve had multiple complaints.

**Table 1:** Incidence of syndrome/symptoms presented by sexually transmitted infections patients

Syndrome/symptoms	Male n = 192 No. (%)	Female n = 108 No. (%)	Total n = 300 No. (%)
GDS*	63 (33)	54 (50)	117 (39)
GUS (total)	61 (32)	30 (27)	91 (30)
Only vesicle	46 (67)	30 (100)	76/91 (84)
Sore/ulcer	15 (25)	0 (0)	15/91 (16)
Anogenital wart	25 (13)	26 (24)	51 (17)
Umbilicated nodule	9 (5)	5 (5)	14 (5)
Macular/papular/maculopapular rash	15 (8)	9 (8)	24 (8)
GDS and GUS	3 (2)	0 (0)	3 (1)
Anogenital wart and maculopapular rash	6 (3)	0 (0)	6 (2)
GDS and anogenital wart	0 (0)	3 (3)	3 (1)

* Vaginal discharge in females without per speculum examination and urethral discharge in males.

### Prevalence of laboratory-confirmed sexually transmitted infection

The prevalence of various STIs along with HIV, HBV, and HCV based on laboratory tests has been shown in Table 2. Genital herpes (IgM HSV-2) accounted for the maximum number of STI (86/300) followed by syphilis (71/300), genital wart (60/300), gonorrhea (58/300), and chlamydial infection (49/300). In all, 35% had more than one STI concomitantly at the time of presentation. The seroprevalence of HIV was 10.3%.

**Table 2:** Laboratory diagnosis, incidence of sexually transmitted infections pathogens

Etiological agent	Male n = 192 No.* (%)	Female n = 108 No.* (%)	Total n = 300 No.* (%)	95% CI
HSV-2	37 (19)	49 (45)	86 (28.7)	23.55-33.79
*T. pallidum*	45 (23)	26 (24)	71 (23.7)	18.86-28.48
HPV	31 (16)	29 (27)	60 (20)	15.47-24.53
*N. gonorrhoeae*	38 (20)	20 (19)	58 (19.3)	14.86-23.8
*C. trachomatis*	28 (15)	21 (19)	49 (16.3)	12.15-20.51
HIV	20 (10)	11 (10)	31 (10.3)	6.89-13.77
HBV	12 (6)	6 (6)	18 (6)	3.31-8.69
*M. contagiosum*	9 (5)	5 (6)	14 (4.7)	2.88-7.06
*T. vaginalis*	0 (0)	14 (13)	14 (4.7)	2.88-7.06
*Candida*	1 (1)	5 (5)	6 (2)	0.42-3.58
HCV	2 (1)	1 (1)	3(1)	–0.13-2.13

* Multiple response.

### Performance of syndromic management against etiological diagnosis of sexually transmitted infection

Table 3 shows the sensitivity, specificity, PPV, and NPV of syndromic management for symptomatic patients coming to the STI clinic. The sensitivity of genital discharge syndrome (GDS) to treat *N. gonorrhoeae* and *C. trachomatis* was 96.5% and 91.8%, respectively. However, the specificity was only 76.3% and 72.5%, respectively. Conversely, the specificity of GDS for the management of *T. vaginalis*, HSV-2, and *Candida* reached 99%, while the sensitivity was 50%, 5.9%, and 50%, respectively.

**Table 3:** Performance of syndromic management

Syndrome and etiology	Laboratory confirmed	Syndromic treatment	St (%)	Sp (%)	PPV (%)	NPV (%)
GDS						
*N. gonorrhoeae*	58	114	96.5	76.3	49.1	98.9
*C. trachomatis*	49	114	91.8	72.5	39.5	72.5
*T. vaginalis*	14	8	50	99.7	87.5	97.6
HSV-2	17	2	5.9	99.6	50	94.6
*Candida*	6	4	50	99.7	75	98.9
GUS						
HSV-2	69	60	82.6	98.7	95	95
Primary syphilis	14	15	81.2	99.2	86.6	98.9

St, sensitivity; Sp, specificity; PPV, positive predictive value; NPV, negative predictive value.

The sensitivity of genital ulcer syndrome for HSV-2 and *T. pallidum* was 82.65% and 81.2%, respectively, while specificity reached to 99% approximately.

The PPV of syndromic management ranged from 75% to 95%, except GDS for *N. gonorrhoeae*, *C. trachomatis*, and HSV-2 where it was 49%, 39%, and 50%, respectively. However, NPV ranges from 94% to 98.9% except GDS for *C. trachomatis* (72.5%).

## DISCUSSION

There is a dearth of information regarding the epidemiology of STIs in India for many reasons such as stigma and discrimination associated with the STI, lack of interdepartmental coordination for studies, poor attendance of STI patients at the public clinics and academic institutions, and availability of limited diagnostic facilities. This in-depth analysis offers an important insight into the burden and pattern of various STIs and on the performance of syndromic management of STIs in comparison with laboratory diagnosis.

In our study, the peak age group of patients ranges from 20 to 30 years (62%), and vast majority of them were male (64%), thus constituting the major bulk of the STI patients. Also, majority of the male patients had promiscuous behavior as 66% of males had more than three sexual partners and 63.9% had contact with CSWs, suggesting that professional prostitution still remains the main source of STI among men having promiscuous behavior.

In our study, GDS was reported in 39% of patients and GUS in 30% while multiple symptoms were seen in 12% of patients. This is a matter of concern in the context of HIV transmission as genital ulcer facilitates the transmission of and enhances susceptibility to HIV infection by sexual contact[19,20] while nonulcerative STIs like gonorrhea and chlamydia increase shedding of the HIV virus in the genital tract by recruiting HIV-infected inflammatory cells as part of normal host response.[19,21]

In the present study, HSV-2 (28.7%) was the commonest infection followed by syphilis (23.7%), wart (20%), gonorrhea (19.3%), chlamydia (16.3%), HIV (10.3%), HBV (6%), *T. vaginalis* (4.7%), *M. contagiosum* (4.7%), *Candida* (2%), and HCV (1%). A marked decline in bacterial STIs, resulting in an apparent increase in viral STIs, has been reported from different regions of India.[7,8,22] Our study confirmed a similar pattern of higher incidence of viral STIs which could be due to the increased usage of antibiotics. Also, a high incidence of HIV seropositivity (10.3%) in the study population indicates the close association of STI with HIV and the importance of early diagnosis of these curable diseases. Previous studies from different parts of the country have also supported these observations.[22]

Algorithms based on a syndromic approach were evaluated in many different settings.[23,24] In our study, the sensitivity of the syndromic approach for the treatment of *N. gonorrhoeae* and *C. trachomatis* was 91.83% and 96.5%, respectively, which was fairly high indicating that large number of those presented with GDS related to gonorrhea and chlamydia were effectively treated. However, their low specificity (72–76%) indicates that many individuals were falsely diagnosed and treated as positive. This overdiagnosis and overtreatment expose more patients to unnecessary antibiotics which could result in the emergence of antimicrobial resistance. For example, over the past decade, strains of *N. gonorrhoeae* have been reported to develop high levels of resistance against several antimicrobial agents, previously used for the treatment of gonorrhea.[25] Indian studies have also reported an increase in the spectrum and level of antibiotic resistance of *N. gonorrhoeae* isolates in the recent year compared to that seen previously.[26]

The present study showed low sensitivity of GDS in detecting *T. vaginalis* (50%), HSV-2 (5.9%), and *Candida* (50%) suggesting that syndromic management for patients with GDS related to these pathogens will not be very useful.

The algorithm used for GUD tries to identify the presence of herpes, syphilis and/or chancroid.[14] In our study population, the sensitivity of GUS for herpes and syphilis was 82.6% and 82.1%, respectively, while the specificity was 98.7% and 99.7%, respectively, suggesting that syndromic management for GUD is not much effective in identifying herpes and syphilis. Various studies have been done to validate diagnostic algorithm for GUS. In a study conducted in China,[27] when syndromic management was used, all patients with syphilis had been correctly treated yielding 100% sensitivity but a large proportion of nonsyphilitic patients were overtreated yielding 5% specificity. In contrast, in a study conducted in the red light area of Surat, India, syndromic management for syphilis yielded 14.8% sensitivity and 96.7% specificity.[28]

In conclusion, viral STIs constitute the major burden of the STI clinic and enhance the susceptibility of an individual to acquire or transmit HIV through sexual contact. Though the syndromic approach has been a major step forward in rationalizing and improving the management of STIs, but syndromic algorithms have some shortcomings, and they need to be periodically reviewed and adapted to the epidemiological patterns of STIs in a given setting.
